# Convolutional Recurrent Neural Network-Based Event Detection in Tunnels Using Multiple Microphones

**DOI:** 10.3390/s19122695

**Published:** 2019-06-14

**Authors:** Nam Kyun Kim, Kwang Myung Jeon, Hong Kook Kim

**Affiliations:** School of Electrical Engineering and Computer Science, Gwangju Institute of Science and Technology (GIST), Gwangju 61005, Korea; skarbs001@gist.ac.kr (N.K.K.); kmjeon@gist.ac.kr (K.M.J.)

**Keywords:** tunnel accident detection, sound event detection (SED), non-negative tensor factorization (NTF), convolutional recurrent neural network (CRNN), online noise learning

## Abstract

This paper proposes a sound event detection (SED) method in tunnels to prevent further uncontrollable accidents. Tunnel accidents are accompanied by crashes and tire skids, which usually produce abnormal sounds. Since the tunnel environment always has a severe level of noise, the detection accuracy can be greatly reduced in the existing methods. To deal with the noise issue in the tunnel environment, the proposed method involves the preprocessing of tunnel acoustic signals and a classifier for detecting acoustic events in tunnels. For preprocessing, a non-negative tensor factorization (NTF) technique is used to separate the acoustic event signal from the noisy signal in the tunnel. In particular, the NTF technique developed in this paper consists of source separation and online noise learning. In other words, the noise basis is adapted by an online noise learning technique for enhancement in adverse noise conditions. Next, a convolutional recurrent neural network (CRNN) is extended to accommodate the contributions of the separated event signal and noise to the event detection; thus, the proposed CRNN is composed of event convolution layers and noise convolution layers in parallel followed by recurrent layers and the output layer. Here, a set of mel-filterbank feature parameters is used as the input features. Evaluations of the proposed method are conducted on two datasets: a publicly available road audio events dataset and a tunnel audio dataset recorded in a real traffic tunnel for six months. In the first evaluation where the background noise is low, the proposed CRNN-based SED method with online noise learning reduces the relative recognition error rate by 56.25% when compared to the conventional CRNN-based method with noise. In the second evaluation, where the tunnel background noise is more severe than in the first evaluation, the proposed CRNN-based SED method yields superior performance when compared to the conventional methods. In particular, it is shown that among all of the compared methods, the proposed method with the online noise learning provides the best recognition rate of 91.07% and reduces the recognition error rates by 47.40% and 28.56% when compared to the Gaussian mixture model (GMM)–hidden Markov model (HMM)-based and conventional CRNN-based SED methods, respectively. The computational complexity measurements also show that the proposed CRNN-based SED method requires a processing time of 599 ms for both the NTF-based source separation with online noise learning and CRNN classification when the tunnel noisy signal is one second long, which implies that the proposed method detects events in real-time.

## 1. Introduction

Recently, millions of sensors have been deployed in almost all urban areas, industrial facilities, and other environments that are rapidly increasing in volume and scope [1]. In practice, monitoring human activities requires a tremendous amount of resources. To this end, research on automated surveillance has progressed rapidly, focusing on video- or image-based approaches operating in real-world environments [2]. However, video data are sometimes unavailable due to poor lighting conditions or the target being out of view of the camera system [3], so analysis based only on visual data is insufficient and error prone [4]. To overcome this disadvantage, different types of sensors have been designed either alone or in conjunction with video signals. For example, ultraviolet/infrared cameras are suitable for detecting fires in tunnels [5], and radar sensors are deployed to monitor vehicles in tunnels [6]. In addition to those sensors, acoustic sensors can support video sensors, resulting in many applications, such as surveillance for intruder detection [7], public environmental monitoring [8], multimedia analysis [9], and speaker position detection [10].

**Figure 1:**
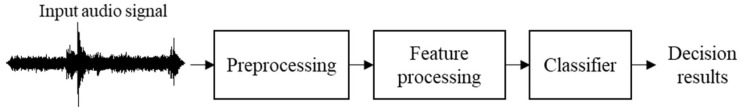
Block diagram of a typical SED method.

Numerous methods dealing with sound event detection (SED), which is defined as the task of identifying the temporal activities of each sound event [11], have been proposed. Figure 1 shows a block diagram of a typical SED method that consists of three processing stages: (1) preprocessing, (2) feature processing, and (3) classification. The first processing stage of SED preprocesses an input sound signal by using the techniques for noise reduction [12] and target audio source separation [13]. The preprocessed sound signal is passed to the second processing stage to extract features for the classification. In particular, the feature extraction is generally related to the type of classifier, which is involved in the third processing stage. Many different types of classifiers are used for SED. Among them, model-based classifiers, such as the support vector machine (SVM) [4] and the hidden Markov model (HMM) [14], have been widely used. In these approaches, a statistical model is trained for each predefined sound event class, and then the onsets and offsets of each sound event are detected using the trained models. Recently, deep neural network-based classifiers, such as the convolutional neural network (CNN) [15] and recurrent neural network (RNN) [16,17], have been proposed. In particular, a classifier based on the convolutional recurrent neural network (CRNN) has been reported to have better classification accuracy than those based on CNN or RNN alone [11,18,19].

As mentioned earlier, SED can be applied to various surveillance systems. Among them, this paper focuses on sound-based accident detection in a tunnel environment. As traffic infrastructure is growing, the number of tunnels has increased. Thus, car accidents in tunnels are expected to be more frequent. In particular, due to the closed spatial characteristic of tunnels, it is vital to detect accidents within them. Moreover, it is necessary to prevent second accidents by detecting first accidents quickly and providing information to the rear vehicles. In order to determine tunnel accidents, the use of video data coming from a closed-circuit television (CCTV) has been popular, but video-based event detection (VED) can often cause false alarms due to various adverse conditions such as darkness, severe weather, a shaking camera, and a limited viewing angle. On the other hand, when a car crash occurs behind the camera, there are no visual data regarding the accident, but the crash sound can be clearly recorded by a microphone [3]. This implies that SED can be replaced with VED in such adverse conditions.

As another example, the 1999 Mont Blanc accident involved a truck that caught on fire while colliding with other vehicles, which took 39 lives [20]. When the accident occurred, the driver abandoned the vehicle and informed the control center via an emergency call. However, the precise location of the accident was not confirmed because the video data from the CCTV could not be identified. The tunnel fire brigade arrived in 57 min, but due to the high-density smoke, it caused many casualties. As illustrated in this case, if a large-scale accident occurs in a tunnel, it can cause large-scale property damage. In addition, it can be difficult to judge the situation of the accident scene due to fire smoke or dust. Therefore, SED-based accident analysis can be applied in such situations, as sound can be captured even in fields obscured by dust and obstacles. Accidents in tunnels are mainly caused by driver error or structural faults, and they can lead to death if they are not responded to quickly enough. In addition, they can progress rapidly from minor accidents to major disasters [5]. Since the processing time for sound data is usually less than that of video data, this makes SED work faster than VED.

**Figure 2:**
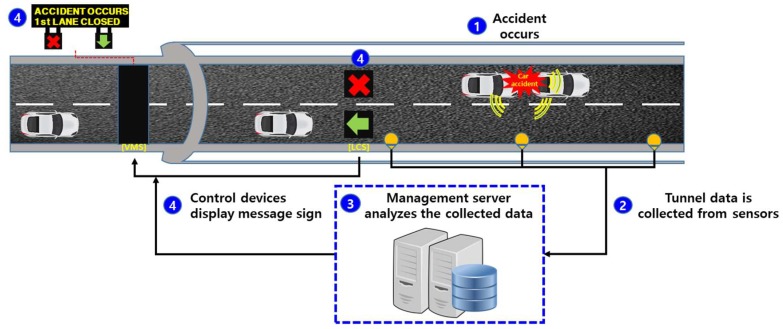
Architecture of an accident management system using acoustic sensors in a tunnel.

Figure 2 shows the architecture of an accident management system in a tunnel [21]. As shown in the figure, when an accident occurs in a tunnel, sensor data are collected from the sensors placed in the tunnel wall, where the sensor data could be video data from the CCTV or sound data from the acoustic sensors or microphone array. Then, the sound data are analyzed in the management server by using an SED algorithm to determine whether an accident has occurred. When an accident is detected, the traffic flow is subsequently managed in a safe and efficient manner using variable message signs (VMSs) and lane control systems (LCSs). In this case, negative effects caused by accidents can be minimized if the processing time of the SED is kept as short as possible.

Several research works have applied SED to accident detection in roads and tunnels [4,21,22,23]. In fact, the preprocessing of some previous methods was done using a non-negative tensor factorization (NTF) technique [21,22], and then the feature parameters were extracted by using signal processing techniques (e.g., the log-mel spectrogram and mel-frequency cepstral coefficient (MFCC) [4]). Recently, deep learning-based feature extraction approaches have been proposed. For example, the feature maps from convolutional layers in a CNN were used for genre classification [24] and audio classification [25]. After that, the classifier was designed based on a statistical approach, such as the SVM [4,23] or the Gaussian mixture model (GMM)–HMM [21,22]. The performance of the previous methods was reasonable when the background noise varied slowly. However, in a rapidly varying noise environment depending on the road or weather conditions, these techniques could increase the errors due to mismatches between the pre-trained noise models and the actual background noise [23]. Therefore, the noise models should be adapted according to the incoming background noise. Moreover, it is known that deep neural network-based approaches outperform statistical approaches in speech and audio processing [26,27,28,29]. Therefore, the classifier also needs to be designed using a deep neural network.

In this paper, a new SED method in tunnels is proposed to prevent uncontrollable further accidents. As shown in Figure 1, the proposed SED method also consists of the preprocessing of tunnel acoustic signals, mel-filterbank analysis, and the classification of acoustic events in tunnels. In the preprocessing stage of the proposed method, an NTF technique [22] is also used to separate both the acoustic event signal and background noise signal from the noisy signal in a tunnel. The reason behind the selection of the NTF technique for separating the event audio and noise from the input noisy signals is motivated by the research conducted on the combination of deep learning and non-negative matrix factorization (NMF) [30,31]. By doing this, noise that is not observed during training can be reduced by the neural networks. Since the SED covered by this paper aims for robust operation in various tunnel noise environments that cannot be handled in the training process, the proposed method also attempts to combine the NMF-based preprocessing and the deep learning-based classifier. Thus, this paper employs the NTF-based sound source separation, which is a generalized form of the NMF for the tensor process, to conduct preprocessing on the multi-channel noisy signal.

Unlike the previous method in [22], the NTF technique used in this paper includes online noise learning so that the separation performance by NTF is improved under adverse tunnel noise conditions. After that, the mel-filterbank parameters are extracted from the separated acoustic event signal and from the separated background noise, respectively, resulting in two sets of mel-filterbank parameters. Next, a CRNN-based classifier is proposed to utilize the two sets of mel-filterbank parameters. The proposed CRNN is composed of two CNNs followed by an RNN with gated recurrent units (GRUs). Here, the two CNNs are one event CNN and one noise CNN, and the event CNN is trained using a set of mel-filterbank parameters from the acoustic event signals separated by NTF, while the noise CNN is trained using those from background noise that is adapted by online noise learning in NTF. Next, the outputs of the two CNNs are concatenated as input features for the following RNN. Finally, the performance of the proposed SED method employing the proposed CRNN and NTF employing online noise learning is evaluated in terms of event detection accuracy, and it is compared with those of a GMM–HMM classifier [22], CNN-based classifier, and conventional CRNN-based classifier [32], where the preprocessing stage is a conventional NTF without any noise adaptation. In addition, the effect of online noise learning on the accuracy of the NTF-based SED is discussed.

The main contributions of this paper are as follows: (1) to incorporate online learning into NTF for tunnel noise estimation, and (2) to model the event sound and noise individually to improve the detection performance. In other words, the noise basis for NTF is adapted with an online noise learning technique to cope with the diverse acoustic environments of the tunnel. In addition, even if the quality of the separated event audio signal is improved by online noise learning, the tunnel noise is further considered in the classification model. To this end, a CRNN-based SED network is designed to have two subnetworks based on multiple CNNs in order to accept the spectra of each separated sound event and background noise.

The remainder of this paper is organized as follows: Section 2 reviews a conventional SED method applied in a tunnel, where NTF and GMM–HMM are used as a preprocessor and a classifier, respectively. Next, Section 3 proposes a new CRNN-based SED method using an online noise learning technique, where the CRNN architecture is newly proposed to accommodate the event signal and background noise with two different CNNs. Section 4 evaluates the detection accuracy of the proposed SED method and compares it with those of statistical approaches using SVM and GMM–HMM as well as other neural network-based approaches. Finally, Section 5 concludes this paper.

## 2. Review of a Conventional SED Method in a Tunnel

**Figure 3:**
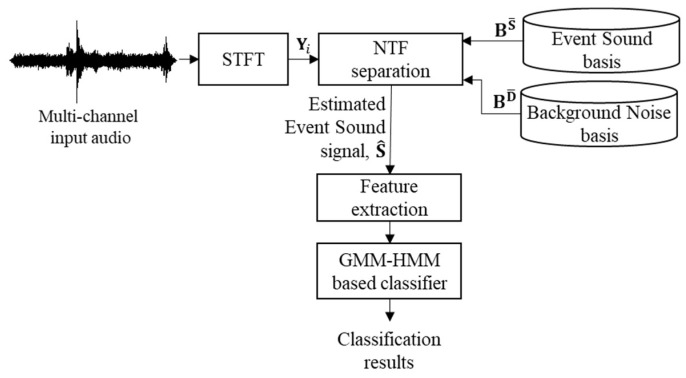
Block diagram of a conventional GMM–HMM-based SED method.

This section describes a GMM–HMM-based SED method using multi-channel signals in a tunnel, as proposed in [22]. Figure 3 shows a block diagram of this conventional method. As shown in the figure, multi-channel noisy signals are captured by multiple microphones and then transformed into the frequency domain by applying a short-time Fourier transform (STFT). Next, an NTF technique is applied to separate the spectrum of an event sound signal from that of the multi-channel noisy signal. Then, the estimated event sound signal is obtained by applying an inverse STFT to the separated spectrum of the event sound signal. After that, feature extraction is performed from the estimated event sound signal, and then a GMM–HMM-based classifier is constructed using the extracted feature parameters.

### 2.1. NTF-Based Source Separation

An NTF-based source separation method attempts to decompose the input noisy signal into the event sound and noise signal [22]. Let $y_{i}^{c}\left(n\right)$ be the noisy signal of the *c*-th channel and the i-th frame. Then, the multi-channel noisy signal at the *i*-th frame, $y_{i}\left(n\right)=\left[y_{i}^{1}\left(n\right),y_{i}^{2}\left(n\right),\;\cdots ,y_{i}^{C}\left(n\right)\right],$ is represented as:

(1) $$y_{i}\left(n\right)=s_{i}\left(n\right)+d_{i}\left(n\right)$$

where C is the number of channels and $s_{i}\left(n\right)\;=\;\left[s_{i}^{1}\left(n\right),s_{i}^{2}\left(n\right),\;\cdots ,s_{i}^{C}\left(n\right)\right]$ and $d_{i}\left(n\right)\;=\;\left[d_{i}^{1}\left(n\right),d_{i}^{2}\left(n\right),\;\cdots ,d_{i}^{C}\left(n\right)\right]$ are the multi-channel clean event sound signal and noise at the *i*-th frame, respectively. After applying a *K*-point STFT to each $y_{i}^{c}\left(n\right)$, its spectrum, $Y_{i}^{c}\left(k\right)$, is concatenated as $Y_{i}\;=\;\left[Y_{i}^{1}\left(k\right),Y_{i}^{2}\left(k\right),\;\cdots ,Y_{i}^{C}\left(k\right)\right]$. Then, the multi-channel event sound spectrum, $S_{i}$, is estimated from $Y_{i}$ by using a supervised NTF-based source separation technique [33].

In the NTF framework, a channel, time, and frequency (CTF) matrix is first constructed by concatenating *M* consecutive $Y_{i}$s as $Y\;=\;\left[Y_{i-M+1},Y_{i-M+2},\cdots ,Y_{i}\right]$, where the dimensions of the CTF matrix are (C × K × M). Note that $Y\;≅\;\hat{S}\;+\hat{D}$ is assumed, where $\hat{S}$ and $\hat{D}$ are the CTF matrices of the estimates of $s_{i}\left(n\right)$ and $d_{i}\left(n\right)$, respectively, because this assumption has provided satisfactory results for NTF-based source separation [33]. Here, a block-wise NTF decomposition is performed as follows [34]:

(2) $$\begin{array}{l} Y=\underset{r\in R_{Y}}{\sum }C_{r}^{Y}⊗B_{r}^{Y}⊗A_{r}^{Y}=\underset{r_{S}\in R_{S},\;r_{D}\in R_{D}}{\sum }\left[C_{r_{S}}^{S}\;C_{r_{D}}^{D}\right]⊗\left[B_{r_{S}}^{S}\;B_{r_{D}}^{D}\right]⊗\left[A_{r_{S}}^{S}\;A_{r_{D}}^{D}\right]\\ \;=\underset{r_{S}\in R_{S}}{\sum }C_{r_{S}}^{S}⊗B_{r_{S}}^{S}⊗A_{r_{S}}^{S}+\underset{r\in R_{D}}{\sum }C_{r_{D}}^{D}⊗B_{r_{D}}^{D}⊗A_{r_{D}}^{D}≅\hat{S}+\hat{D} \end{array}$$

where ⊗ refers to the tensor product and $C_{r}^{X}$, $B_{r}^{X}$, and $A_{r}^{X}$ are the channel gain matrix, basis matrix, and activation matrix of a CTF matrix, X, with a rank of *r*, respectively. In this case, X could be **Y**, **S**, or **D**, and $C_{r}^{Y}\;=\;\left[C_{r_{S}}^{S}\;C_{r_{D}}^{D}\right]$, $B_{r}^{Y}\;=\;\left[B_{r_{S}}^{S}\;B_{r_{D}}^{D}\right],$ and $A_{r}^{Y}\;=\;\left[A_{r_{S}}^{S}\;A_{r_{D}}^{D}\right]$. Additionally, $R_{S}\;$ and $\;R_{D}$ ($R_{Y}\;=R_{S}\;+\;R_{D}$) are the ranks of the basis matrices for S and D, respectively. In addition, $C_{r_{S}}^{S}$, $B_{r_{S}}^{S}$, and $A_{r_{S}}^{S}$ are the $r_{S}$-th column vectors of the ($C\;\times \;R_{S}$)-dimensional channel gain matrix, ($K\;\times \;R_{S}$)-dimensional basis matrix, and ($M\;\times \;R_{S}$)-dimensional activation matrix, respectively. $C_{r_{D}}^{D}$, $B_{r_{D}}^{D}$, and $A_{r_{D}}^{D}$ are also defined similarly to $C_{r_{S}}^{S}$, $B_{r_{S}}^{S}$, and $A_{r_{S}}^{S}$, respectively. As described in Equation (2), $\hat{S}$ and $\hat{D}$ are obtained after estimating $C_{r_{S}}^{S}$, $B_{r_{S}}^{S}$, $A_{r_{S}}^{S}$, $C_{r_{D}}^{D}$, $B_{r_{D}}^{D}$, and $A_{r_{D}}^{D}$ for all ranks, $r_{S}$ and $r_{D}$, by using the NTF technique.

As shown in Figure 3, the conventional NTF technique described in [22] pre-trains the event sound basis matrices and background noise basis matrices, $B_{r_{S}}^{S}\;$ and $\;B_{r_{D}}^{D}$, from the previously prepared clean event sound signal and noise database, respectively. The procedure of basis estimation is described in [34]. Next, the NTF-based source separation method is performed to estimate $C_{r_{S}}^{S}$, $B_{r_{S}}^{S}$, and $A_{r_{S}}^{S}$ for $B_{r_{S}}^{S}$ and $C_{r_{D}}^{D}$, $B_{r_{D}}^{D}$, and $A_{r_{D}}^{D}$ for $B_{r_{D}}^{D}$. Then, the channel gain and activation matrices at the *i*-th frame are iteratively estimated using the following equations:

(3) $$\;\left[{\hat{C}}_{l,i;r_{S},c}^{S}\;{\hat{C}}_{l,i;r_{D},c}^{D}\right]=\left[{\hat{C}}_{l-1,i;r_{S},c}^{S}\;{\hat{C}}_{l-1,i,r_{D},c}^{D}\right]\circ \frac{\sum_{k\in K,m\in M}P_{l-1,i;c,k,m}\left[B_{r_{S},k}^{S}B_{r_{D},k}^{D}\right]\left[{\hat{A}}_{l-1,i;r_{S},m}^{S}{\hat{A}}_{l-1,i;r_{D},m}^{D}\right]}{\sum_{k\in K,m\in M}\left[B_{r_{S},k}^{S}B_{r_{D},k}^{D}\right]\left[{\hat{A}}_{l-1,i;r_{S},m}^{S}{\hat{A}}_{l-1,i;r_{D},m}^{D}\right]},$$

(4) $$\left[{\hat{A}}_{l,i;r_{S},m}^{S}\;{\hat{A}}_{l,i;r_{D},m}^{D}]=\;[{\hat{A}}_{l-1,i;r_{S},m}^{S}\;{\hat{A}}_{l-1,i;r_{D},m}^{D}\right]\circ \frac{\sum_{c\in C,k\in K}P_{l-1,i;c,k,m}\left[{\hat{C}}_{l-1,i;r_{S},c}^{S}{\hat{C}}_{l-1,i;r_{D},c}^{D}\right]\left[B_{r_{S},k}^{S}B_{r_{D},k}^{D}\right]}{\sum_{c\in C,k\in K}\left[{\hat{C}}_{l-1,i;r_{S},c}^{S}{\hat{C}}_{l-1,i;r_{D},c}^{D}\right]\left[B_{r_{S},k}^{S}B_{r_{D},k}^{D}\right]},$$

(5) $${\hat{Y}}_{l,i;c,k,m}=\sum_{r_{S}\in R_{S}}{\hat{C}}_{l,i;r_{S},c}^{S}⊗B_{r_{S},k}^{S}⊗{\hat{A}}_{l,i;r_{S},m}^{S}+\sum_{r\in R_{D}}{\hat{C}}_{l,i;r_{D},c}^{D}⊗B_{r_{D},k}^{D}⊗{\hat{A}}_{l,i;r_{D},m}^{D}$$

where multiplication (∘) and division are applied on an element-by-element basis and *l* is an iteration index. In addition, $P_{l,i;c,k,m}\;=\;Y_{\left(i;c,k,m\right)}/{\hat{Y}}_{\left(l,i;c,k,m\right)\;}.\;$ Note that $X_{l,i;r,e}$ is the *e*-element of the *r*-th column vector of X at the *i*-th frame for the *l*-th iteration. Equations (3)–(5) are terminated when the relative reduction of the Kullback–Leibler (KL) divergence between iterations *l* and (*l*−1) is less than a predefined threshold [34]. Note that all elements of the matrices, $C_{r_{S}}^{S}$, $A_{r_{S}}^{S}$, $C_{r_{D}}^{D}$, and $A_{r_{D}}^{D}$**,** are initialized by setting a random value between 0 and 1. Finally, the multi-channel event sound signals at the *i*-th frame are obtained by using the equation of ${\hat{S}}_{i}\;=\;\underset{r_{S}\in R_{S}}{\sum }{\hat{C}}_{L,i;r_{S}}^{S}⊗B_{r_{S}}^{S}⊗{\hat{A}}_{L,i;r_{S}}^{S}$**,** followed by an inverse STFT where the iteration is finished at *L*.

The NTF-based source separation employed in the conventional SED method works well when the training and test noise conditions are matched. However, the noise basis could be inadequate when tunnel acoustic environments differ from those in the noise database. This is because it pre-trains noise basis matrices from a noise database recorded in tunnel environments. Thus, the noise basis should be updated adaptively to the environment where the SED method is implemented.

### 2.2. GMM–HMM-Based Classification

HMM has been widely used as a typical probabilistic method in modeling time series data such as speech, audio, and even image data [35]. In [22], an HMM was applied to classify event sounds in a tunnel for SED. To extract acoustic feature parameters, the event sound signal separated by the NTF technique was segmented into consecutive frames of 4096 samples with 50% overlap between frames at a 48-kHz sampling rate. Then, a 4096-point fast Fourier transform (FFT) was applied after multiplying each frame by a Hamming window. The spectrum was used to extract 20 MFCCs [36], and then their delta and delta–delta parameters were concatenated to make a 60-dimensional feature vector per frame. In this conventional method, two classes of possible sound events in tunnels were considered: car crash and tire skid. Then, each event sound class and background noise was modeled by a five-state left-to-right HMM that consisted of a total of 200 GMMs, where the Gaussian mixtures were used in modeling the probability density functions of observations in each state. Finally, each HMM was trained using the MFCC features extracted from the corresponding event sound signals or noise.

**Figure 4:**
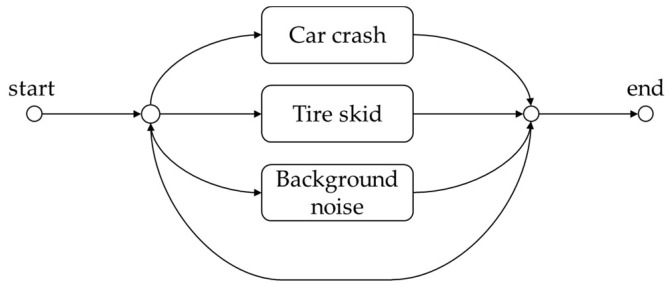
The network architecture of sound event classification based on GMM–HMM.

Figure 4 illustrates the network architecture for sound event classification based on GMM–HMM, where each event including background noise is represented by a GMM–HMM as described above. In other words, the test signal recorded from a tunnel is processed by the NTF source separation and feature extraction, and then the MFCC feature parameters of the test signal are passed into the network to calculate the likelihood of each HMM by using the Viterbi algorithm [37]. Finally, the event class is selected as the HMM giving the maximum likelihood.

Although the GMM–HMM-based classifier has been widely used in various acoustic event classification tasks, recent research has shown that deep neural network-based models with CNN or RNN architectures are more accurate than GMM–HMM-based methods when performing the same tasks [11]. For this reason, the following section proposes a new method that improves the accuracy by performing online noise learning in the NTF framework as well as by proposing a deep neural network-based model using a CRNN.

## 3. Proposed CRNN-Based SED Method

**Figure 5:**
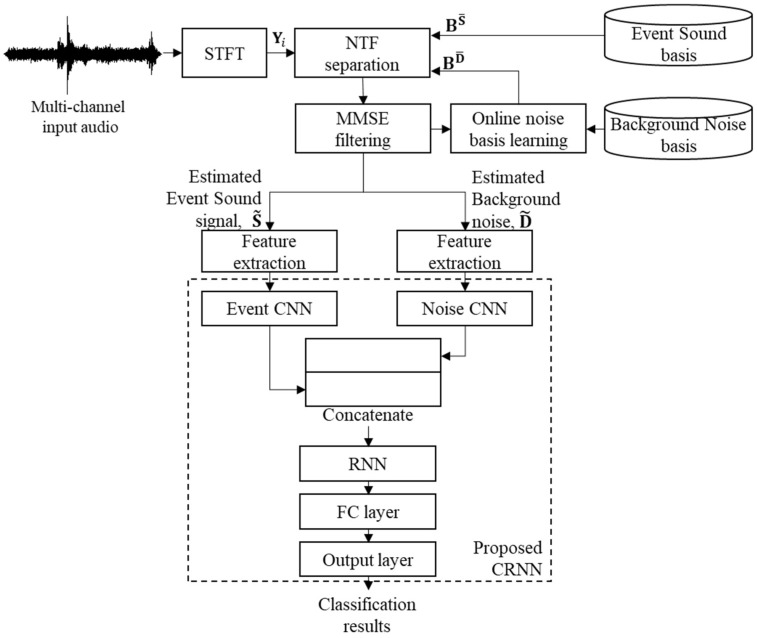
Block diagram of the proposed SED method based on the NTF source separation with online noise learning and a CRNN-based classifier with event sound and noise CNNs.

This section proposes a new SED method for tunnel event sound detection, as shown in Figure 5. Compared with Figure 3, the proposed SED method is characterized by the online noise learning for the NTF-based source separation and the CRNN-based classification, which will be described in the following subsections.

### 3.1. NTF-Based Source Separation with Online Noise Learning

In order to cope with the diverse acoustic environments of tunnels, the noise basis should be adapted with an online noise learning technique to improve the performance of the source separation in adverse noise conditions. First, the conventional NTF is performed on the input tunnel noisy signal at the *i*-th frame, $y_{i}\left(n\right)$, as described in Section 2.1. In this case, the noise basis matrix, $B_{r_{D}}^{D},\;$ is replaced with the noise basis matrix updated in the (*i*−1)-th frame, $B_{i-1;r_{D}}^{D},\;$ by the procedure described below. That is, the channel gain matrix, ${\hat{C}}_{L,i;r_{S}}^{S},\;$ and activation matrix, ${\hat{A}}_{L,i;r_{S}}^{S}$, for the event sound are estimated after applying the iterations of Equations (3)–(5). Note here that the ranks of event sound and noise bases $R_{S}$ and $R_{D}$ are set to 100 from preliminary experiments. Then, the spectral magnitudes of the event sound and noise, ${\hat{S}}_{i}$ and ${\hat{D}}_{i}$, respectively, are estimated using the following equations:

(6) $${\hat{S}}_{i}=\underset{r_{S}\in R_{S}}{\sum }{\hat{C}}_{L,i;r_{S}}^{S}⊗B_{r_{S}}^{S}⊗{\hat{A}}_{L,i;r_{S}}^{S},$$

(7) $${\hat{D}}_{i}=\underset{r_{D}\in R_{D}}{\sum }{\hat{C}}_{L,i;r_{D}}^{D}⊗B_{i-1;r_{D}}^{D}⊗{\hat{A}}_{L,i;r_{D}}^{D}.$$

Next, the noise basis matrix, $B_{i-1;r_{D}}^{D},$ is updated from ${\hat{S}}_{i}$ and ${\hat{D}}_{i}.$ In this paper, only one channel signal is used for the online noise learning instead of using multi-channel signals. To this end, the channel that is the most suitable for the noise update should be selected. With the help of the estimated channel gain, ${\hat{C}}_{L,i;r_{S}}^{S},$ the channel that provides the largest channel gain is selected as:

(8) $${\hat{c}}_{i}=\underset{c\in C}{\mathrm{argmax}}\left[\underset{r_{S}\in R_{S}}{\sum }{\hat{C}}_{L,i;r_{S},c}^{S}\right].$$

Then, the noise spectrum of the *c*-th channel at the *i*-th frame, ${\hat{D}}_{i;{\hat{c}}_{i}},\;$ can be used for the noise update. However, ${\hat{D}}_{i;{\hat{c}}_{i}}$ is the noise estimated only from the noisy signal of the *i*-th frame; thus, it does not consider the noise variation over several frames, which causes misadjusting noise because the noise update is done for the next frame. Thus, instead of directly using ${\hat{D}}_{i;{\hat{c}}_{i}}$, an additional filtering process is designed here to take into account such noise variation. Similar to [38], a minimum mean squared error (MMSE) filter is constructed to obtain noise components for online noise learning.

The MMSE filter has a form of $g_{i}\;=\;\xi_{i}/\left(\xi_{i}+1\right)$, where $\xi_{i}$ is the a priori signal-to-noise ratio (SNR) of the *i*-th frame. In this paper, $\xi_{i}$ is estimated in a decision-directed approach [39] as follows:

(9) $$\xi_{i}=\frac{\alpha {\tilde{S}}_{i-1;{\hat{c}}_{i-1}}+\left(1-\alpha \right){\hat{S}}_{i;{\hat{c}}_{i}}}{\gamma {\tilde{D}}_{i-1;{\hat{c}}_{i-1}}+\beta_{i}\left(1-\gamma \right){\hat{D}}_{i;{\hat{c}}_{i}}}$$

where ${\tilde{S}}_{i-1;{\hat{c}}_{i-1}}\;$ and ${\tilde{D}}_{i-1;{\hat{c}}_{i-1}}$ are the estimates of the event sound and noise at the (*i*−1)-th frame by applying ${\tilde{S}}_{i-1;{\hat{c}}_{i-1}}\;=\;g_{i-1}\circ Y_{i-1;{\hat{c}}_{i-1}}$ and ${\tilde{D}}_{i-1;{\hat{c}}_{i-1}}\;=\;\left(1-g_{i-1}\right)\circ Y_{i-1;{\hat{c}}_{i-1}},\;$ respectively. In Equation (9), α and γ are smoothing coefficients for the sound event and background noise, respectively, and they are set as α = 0.1 and γ = 0.01 through exhaustive experiments. In addition, $\beta_{i}$ is a frame-dependent adaptive noise flooring factor that can be derived from the ratio between the activations of noise and event sound, such that:

(10) $$\beta_{i}=20\log_{10}\frac{\sum_{\;r_{D}\in R_{D}}{\hat{A}}_{i:r_{D}}^{D}/R_{D}}{\sum_{r_{S}\in R_{S}}{\hat{A}}_{i:r_{S}}^{S}/R_{S}}.$$

Note that $\beta_{i}$ reflects the overall SNR of the multi-channel event sound and noise signals because the activation matrices are estimated without regard to any specific channel. After constructing $g_{i}$, the spectral magnitudes of event sound, ${\tilde{S}}_{i;{\hat{c}}_{i}},$ and noise, ${\tilde{D}}_{i;{\hat{c}}_{i}},\;$ are estimated again using the following equations:

(11) $${\tilde{S}}_{i;{\hat{c}}_{i}}\;=\;g_{i}\circ Y_{i;{\hat{c}}_{i}}\;\mathrm{and}\;{\tilde{D}}_{i;{\hat{c}}_{i}}=\left(1-g_{i}\right)\circ Y_{i;{\hat{c}}_{i}}$$

where multiplication (∘) is applied on an element-by-element basis

Next, M  frames of ${\tilde{D}}_{i;{\hat{c}}_{i}}$ are also concatenated as ${\tilde{D}}_{i}\;=\;\left[{\tilde{D}}_{i-M+1;{\hat{c}}_{i}},\cdots ,{\tilde{D}}_{i;{\hat{c}}_{i}}\right]$ to apply a discriminative dictionary learning technique [38] such as:

(12) $${\tilde{B}}_{l,i}^{D}={\tilde{B}}_{l-1,i}^{D}\;\circ \frac{\left(\frac{{\tilde{D}}_{i}}{{\tilde{B}}_{l-1,i}^{D}\;{\hat{A}}_{L,i}^{D}}\right){\left({\hat{A}}_{L,i}^{D}\right)}^{T}}{1{\left({\hat{A}}_{L,i}^{D}\right)}^{T}}$$

where *T* is the transpose operation and the basis matrix for the update is initialized as ${\tilde{B}}_{0,i}^{D}=B_{i}^{D}.$ Additionally, the noise basis matrix, ${\tilde{B}}_{l,i;r_{D}}^{D},$ is iteratively updated by minimizing the KL divergence. However, the update in Equation (12) is performed for all ranks of the basis matrix, which causes excessive updating of the noise basis matrix even when the event sound signal is dominant. To prevent this problem, a noise basis to be updated that satisfies the following equation is selected:

(13) $$I_{i}\left(r\right)=\left\{r|\frac{1}{M}\overset{i}{\underset{\;j=i-M+1}{\sum }}{\hat{A}}_{j;r}^{D}>\eta \right\}$$

where $\eta \;=\;1/\left(M\cdot R_{S}\right)\overset{i}{\underset{\;j=i-M+1}{\sum }}\overset{R_{S}}{\underset{r=1}{\sum }}{\hat{A}}_{L,j;r}^{S},$ and $I_{i}\left(r\right)\;=\;1$ means that the *r*-th basis should be updated to accommodate the noise that appears at the i-th frame. Then, the activation matrix, ${\hat{A}}_{L,i}^{D},$ is decomposed into ${\hat{A}}_{i;r_{D}\in I_{i;u}}^{D}$ and ${\hat{A}}_{i;r_{D}\in I_{i;f}}^{D}$**,** where $I_{i;u}\;=\;\left\{r|I_{i}\left(r\right)\;=\;1\right\}$ and $I_{i;f}\;=\;\left\{r|I_{i}\left(r\right)\;=\;0\right\},$ respectively. After that, Equation (12) is modified as:

(14) $${\tilde{B}}_{l,i;r_{D}\in I_{i;u}}^{D}\;=\;{\tilde{B}}_{l-1,i;r_{D}\in I_{i;u}}^{D}\;\circ \left(\left(\frac{{\tilde{D}}_{i}}{{\tilde{B}}_{l-1,i;r_{D}\in I_{i;u}}^{D}\;{\hat{A}}_{L,i;r_{D}\in I_{i;u}}^{D}}\right){\left({\hat{A}}_{L,i;r_{D}\in I_{i;u}}^{D}\right)}^{T}/1{\left({\hat{A}}_{L,i;r_{D}\in I_{i;u}}^{D}\right)}^{T}\right)$$

Finally, the basis matrix for the next frame, $B_{i+1}^{D},$ is obtained by concatenating the fixed noise basis $B_{i;r_{D}\in I_{i;f}}^{D}$ and the converged $\;{\tilde{B}}_{i;r_{D}\in I_{i;u}}^{D}$ in Equation (14) as $B_{i+1}^{D}\;=\;\left[B_{i;r_{D}\in I_{i;f}}^{D}\;{\tilde{B}}_{i;r_{D}\in I_{i;u}}^{D}\right]$. Moreover, the spectral magnitudes, ${\tilde{S}}_{i;{\hat{c}}_{i}}$ and ${\tilde{D}}_{i;{\hat{c}}_{i}},\;$ are used as the input for the CRNN-based classifier, which will be explained in the next subsection.

### 3.2. CRNN-Based Event Classification

The CRNN was successfully used in an audio classification task [11], where audio event signals came from a home or residential area and they were modeled only by a neural network without considering the background noise in the model. However, as mentioned earlier, the tunnel environment is more severe than the home or street environments in [11]. Thus, the tunnel noise also needs to be considered in the classification model. To this end, the conventional CRNN architecture is extended here to accommodate the event sound and noise signal together, as shown in the lower part of Figure 5. In other words, the proposed CRNN-based classifier first consists of two CNNs: one event CNN and one noise CNN. Then, the outputs from the two CNNs are concatenated so that the concatenated output in the time and feature dimension is used as the input to an RNN layer. Next, the RNN output is flattened by a fully connected (FC) layer, and then the FC layer is connected to the output layer to classify the event sound or noise.

**Figure 6:**
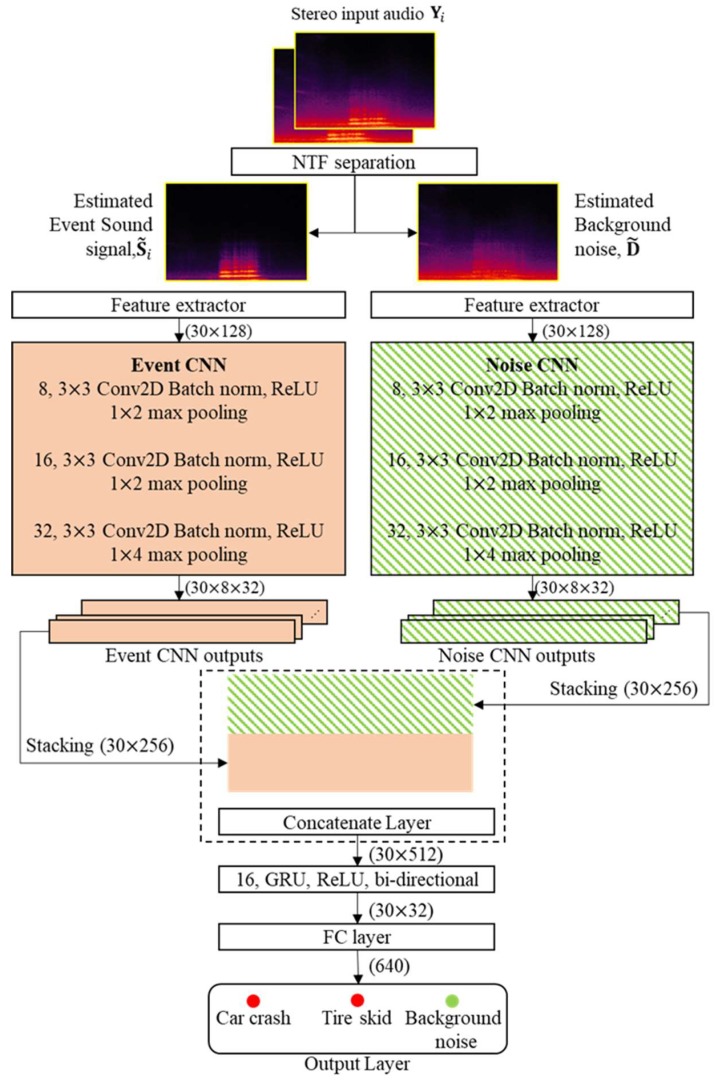
Architecture of the CRNN in the proposed SED method.

Figure 6 illustrates the proposed CRNN-based classifier in detail. First, the stereo-channel input tunnel noisy signal is separated into both event sound signals and background noise using the NTF technique with online noise learning, as described in Section 3.1. Similar to the conventional GMM–HMM-based SED method, each separated signal is sampled at 48 kHz and segmented into consecutive frames of 4096 samples with 50% overlap between the frames. Then, a 4096-point FFT is applied to each separated signal, and a 128-dimensional mel-filterbank analysis [40] is performed for each frame. As an input feature to CNNs, the frames are integrated in 30-frame groups, resulting in a (30 × 128) image. As mentioned previously, each event sound and noise is modeled by a separate CNN, and both CNNs are composed of three convolution layers, where the number of kernels is 8, 16, and 32 for each convolution layer; however, (3 × 3) kernels are all used with a stride size of 2. Moreover, each convolution layer is followed by batch normalization [41], rectified linear unit (ReLU) activation, and a dropout layer [42] with a rate of 0.2 and a (3 × 3) max pooling layer [43]. In particular, the stride size of the max pooling layer is set to (1 × 2) for the first two convolution layers and to (1 × 4) for the third one.

By following the procedure described above, there are two CNN outputs from the event and noise CNN with dimensions of (30 × 8 × 32). Then, they are each stacked into a (30 × 256)-dimensional image and concatenated to construct a (30 × 512)-dimensional image. Next, a bi-directional RNN with 16 GRUs is followed by the concatenated layer in order to learn the temporal context information, where a ReLU is used as an activation function for each GRU. The output of the RNN is inputted to an FC layer with dimensions of (30 × 32). Finally, the output layer with a softmax activation function is used to classify the input tunnel signal as “car crash”, “tire skid”, or background noise.

## 4. Performance Evaluation

The performance of the proposed SED method was evaluated on two different datasets: one was the MIVIA road audio events dataset for publicly available for road surveillance applications [4], and the other dataset was newly organized for SED in tunnel environments. In particular, the latter dataset included artificially generated sound clips as well as sound clips recorded in actual tunnels to compensate for the lack of recording data to train the model parameters of each classifier due to the low frequency of accidents in real tunnels. For the comparison with the proposed method, conventional classifiers including SVM [4,23], GMM–HMM [22], CNN, and a conventional CRNN [32] were evaluated as well as the proposed method. Moreover, the effectiveness of the proposed NTF-based online noise learning for SED in a tunnel environment was examined. In addition, the performance contribution of the mel-filterbanks extracted from the proposed NTF-based online noise learning was compared with those of CNN-based features extracted from both noisy input and NTF without online noise learning. Finally, the computational complexity of the conventional and the proposed SED methods was compared.

### 4.1. Datasets

Table 1 describes the MIVIA road audio events dataset. As shown in the table, it was composed of two events (tire skid and car crash) of 200 audio clips each, whose total length was 326.38 s and 522.6 s for tire skid and car crash, respectively. In addition to the event sounds, the dataset included background noise (2732 s long). All clips were recorded with an omni-directional microphone with a sampling rate of 32 kHz and then up-sampled to 48 kHz. Since this dataset was recorded by a single microphone, both the conventional and the proposed SED methods discussed in Section 2 and Section 3 were performed with *C* = 1.

For the evaluation regarding this dataset, four-fold cross-validations were performed, and final outcomes were measured by averaging all cross-validations. In other words, event sounds for tire skid (TS) and car crash (CC) were grouped into four groups, where each group was composed of 50 event clips per event, resulting in 100 clips in total. In addition, the background noise (BN) was divided into four groups so that the length of each group for BN was about 700 s long. After that, three out of the four groups were used together to train the GMM–HMM or neural networks, and the remaining group was used for testing them. Note here that any event clip or noise used in the training was not overlapped with those in the test. This cross-validation was repeated four times.

In order to organize the audio dataset for SED in the tunnel, audio signals were recorded inside an actual 700-m-long one-way tunnel. To record inside the tunnel, an audio recording device with two omni-directional microphones with a distance between the microphones of 14.8 cm apart was installed in the tunnel’s sidewall 500 m away from the entrance. The recording continued for six months. Then, all of the recorded data were split into a training dataset and a test dataset according to the time at which the data were recorded. That is, 84 event clips for the training set and 48 event clips for the evaluation set were excerpted from the audio dataset recorded during the first three months and the remaining three months, respectively. Note here that any sound source used in the evaluation did not belong to the training set.

Despite the long recording time, the number of event clips was not sufficient to train the classifier of SED methods due to the low frequency of accidents in tunnels. For this reason, additional event clips were artificially generated by simulating the tunnel environment.

To generate the simulated data, sound clips of 311 tire skids and 93 car crashes were collected from a Sound-Ideas sound effect dataset [44]. Next, they were artificially distorted by convolving with a room impulse response (RIR) that was modeled by an arch-shaped space based on the Enhanced Acoustic Simulator for Engineers (EASE) [45]. Here, the room parameters designed for the RIR coefficients were set to reflect the structural characteristics of the tunnel where the recording was conducted. In addition, the recorded background noise was mixed with the distorted event sounds to simulate interferences by them. Table 2 shows the number and duration of the collected event sound clips and background noises. Note that the evaluation set contained tire skids or car crashes that appeared just once for an hour of background noise on average, resulting in 48 h of background noise containing 48 sound events.

### 4.2. Neural Network Modeling and Performance Measurement Metrics

The proposed CRNN-based SED method was compared with the CNN and CRNN [32]. Table 3 describes the architectures of the neural networks in detail. All of the neural networks were implemented in the deep learning package Keras (version 2.1.5) [46] using Tensorflow (version 1.5.0). To train the CNN, CRNN, and proposed CRNN, the model weight parameters were initialized by using a zero-mean Gaussian distribution [47]. In addition, each neural network was trained with the mini-batch-wise adaptive moment estimation (ADAM) optimization algorithm to minimize the categorical cross-entropy criterion [48]. For training validation, 10% of the training data were prepared as validation data. The early stopping rule [46] was also applied to terminate the model training with the minimum number of epochs set to 30.

For objective performance evaluation, four different metrics were used as in [4]:

(1) The recognition rate (RR) or the true positive rate (TPR): the rate of correctly classified events of interest;

(2) The false positive rate (FPR): the rate of wrongly classified events of interest when only background sound was present;

(3) The missed detection rate (MDR): the rate of undetected events; and

(4) The area under the receiver operating characteristic (ROC) curve (AUC).

The ROC curve was a plot of the tradeoff between the TPR and FPR of a classifier when its discrimination threshold was varied. The closer an ROC curve was to the top-left corner of the plane, the better the performance. Thus, the AUC should be equal to 1 in a perfect classifier.

### 4.3. Performance Comparison Using the MIVIA Road Audio Events Dataset

This section compares the results of the proposed SED method with those of conventional SED methods applied to the task of analyzing the MIVIA road audio events dataset. First, the conventional methods evaluated here were all based on an SVM classifier using different feature parameters such as MFCC features based on the bag-of-words (BoW) approach [4], temporal and spectral features [4], and selected time and frequency features [23]. Next, audio event classifiers based on GMM–HMM [22], CNN, and CRNN [32] were also evaluated. For these three methods, mel-filterbanks of {$\hat{S}$} in Equation (6) were commonly employed. After that, the SED method using the proposed CRNN architecture with mel-filterbanks of {$\tilde{S}$,$\tilde{D}$} in Equation (11), which were obtained from the online noise learning, was compared with other conventional methods.

Table 4 compares the performances of the SED methods evaluated on the MIVIA road audio events dataset. In the case of the SVM-based SED method, the SVM classifier using the selected time and frequency features [23] outperformed the other two SVM classifiers by achieving an average RR of 95.00%. On the one hand, the conventional GMM–HMM, CNN, and CRNN classifiers were evaluated by using the mel-filterbanks of noisy signal {Y}. As shown in the second row of the table, GMM–HMM showed the worst performance in all measurements. This was because the background noise was not adequately modeled by GMM–HMM. On the other hand, CNN and CRNN showed superior performance compared to GMM–HMM because the learning from large data helped them deal with background noise. Next, in order to investigate the effect of NTF sound source separation on the detection performance, the three classifiers were also applied to the mel-filterbanks of the separated signal {$\hat{S}$}. As shown in the third row of the table, the performance of GMM–HMM was greatly improved because the background noise was effectively reduced by the NTF technique and thus the event sound could be better recognized than GMM–HMM with the mel-filterbanks from the noisy signal. However, the performances of CNN and CRNN were similar to those when the NTF-based sound source separation was not applied. Next, the NTF source separation with online noise learning was applied to the tunnel input noisy signal, and then GMM–HMM, CNN, and CRNN were constructed using the mel-filterbanks of the separated event sound from the NTF with online noise learning {$\tilde{S}$}. However, the performance improvement for all classifiers was marginal, because the level of background noise was relatively low in this dataset.

Finally, the proposed CRNN-based SED method was applied to the mel-filterbanks of the separated signal and noise {$\tilde{S}$,$\tilde{D}$}. Consequently, it was shown from the last row of the table that the proposed method gave the highest RR of 98.25%, the lowest MDR of 1.00%, and the highest AUC of 98.39%, while the FPR was comparable to that of the CNN. This was achieved due to the two CNNs of the proposed CRNN for modeling the event sound and noise separately.

**Figure 7:**
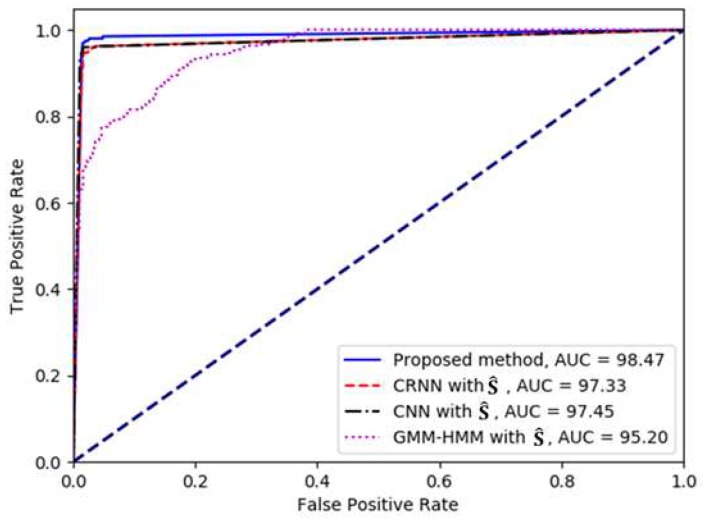
Comparison of the receiver operating characteristic (ROC) curves between the proposed CRNN-based SED method and the other SED methods.

Next, the experimental results of the proposed method were compared with those of the conventional methods based on GMM–HMM, CNN, and CRNN by analyzing the ROC curves, as shown in Figure 7. The ROC curves for the deep neural network-based methods were drawn by obtaining the TPR and FPR according to the different decision thresholds that were applied for the event detection from the softmax probability value. Note that in the case of GMM–HMM, different decision thresholds were applied to the Viterbi score of the HMM. As shown in the figure, the proposed CRNN-based SED method performed better, as the corresponding curve lay closer to the left and top borders of the quadrant than those of the other methods.

### 4.4. Performance Evaluation in a Tunnel Environment

In this subsection, the performance of the proposed SED method was evaluated on the evaluation dataset that was actually recorded inside a tunnel. Moreover, the effectiveness of the NTF-based source separation with online noise learning on the various SED methods including the proposed one was also examined. To this end, each classifier was trained by the tunnel sound event dataset explained in Section 4.1. Moreover, 48 h of the evaluation dataset containing 48 sound events were applied to the SED methods to evaluate their classification accuracy in a real tunnel environment.

Table 5 shows the results of the conventional SED methods and the proposed one before and after the NTF-based source separation with or without online noise learning. Similar to Table 4, in order to examine the effectiveness of NTF on detection performance, GMM–HMM, CNN, and the conventional CRNN-based SED methods were trained using the mel-filterbanks from the noisy spectrum {Y} or the mel-filterbanks of the separated event sound from the NTF {$\hat{S}$}. As shown in the first and second rows of the table, the GMM–HMM with {$\hat{S}$} gave a similar RR and MDR to the GMM–HMM with {Y}, while the former significantly reduced the FPR. On the other hand, CNN and CRNN, after applying the NTF-based sound source separation, showed better performance on RR, MDR, and AUC than those before applying NTF. This was because the NTF-based source separation played a main role in dealing with tunnel background noise. However, their FPRs were increased when compared to those before applying NTF. This was because the CNN and CRNN were trained using only the separated event audio without any consideration of the background noise. Next, the effect of online noise learning was examined by constructing the GMM–HMM, CNN, and CRNN using the mel-filterbanks of the separated event sound from the NTF with online noise learning {$\tilde{S}$}. As shown in the third row of the table, the performances of all classifiers were improved when compared with those using NTF without online noise learning {$\hat{S}$}, except for the FPR of GMM–HMM. Instead, the MDR of GMM–HMM was greatly decreased. Such performance improvement indicated that the online noise learning could influence noise reduction in the separated event sound.

Finally, the performance of the proposed CRNN-based SED method was evaluated using the mel-filterbanks from NTF with online noise learning {$\tilde{S}$,$\tilde{D}$}. As shown in the last row of the table, the proposed CRNN outperformed the other comparatives in all measurements by large margins. In particular, it reduced the recognition error rates by 47.40% and 28.56% when compared to the GMM–HMM-based and the conventional CRNN-based SED methods, respectively. Moreover, the FPR of the proposed CRNN was the lowest among all classifiers, which implied that the two CNNs for the event audio and noise mostly contributed to the detection accuracy under severe tunnel noise conditions, resulting in the highest RR and the lowest FPR.

### 4.5. Performance Comparison of Signal Processing-Based and Deep Learning-Based Features

**Figure 8:**
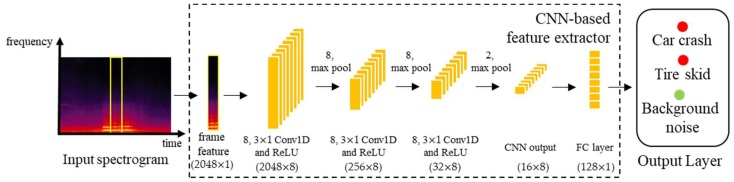
Architecture of the CNN-based feature extractor.

The mel-filterbanks used in this paper were extracted from a signal processing technique. However, as mentioned in Section 1, feature extraction approaches based on deep neural networks have been proposed [49]. Figure 8 shows a block diagram of the CNN-based feature extraction method. As described in Section 3.2, a 4096-point FFT was applied to each frame, and then the spectral magnitudes at 2048 frequency bins were used as an input feature to the CNN. The CNN for feature extraction was composed of three one-dimensional convolutional layers with eight kernels each, where each convolution layer was followed by the ReLU activation and a max pooling layer whose filter size was differently set to 8, 8, and 2 for each convolutional layer. Consequently, a (16 × 8)-dimensional feature map was constructed, and it was flattened by an FC layer to construct the 128-dimensional feature parameters once every frame. Note here that the class (TS, CC, or BN) was presented as a target value to the output layer of this CNN-based feature extraction, and this output layer was removed after extracting the feature. The feature parameters from the CNN were then brought to the input for the CNN-based and CRNN-based classifier described in Table 3.

Table 6 compares the performances of the SED methods evaluated on the MIVIA road audio events dataset, where the CNN-based feature parameters and the mel-filterbanks were extracted from both noisy signal {Y} and the separated event sound from the NTF without online noise learning {$\hat{S}$}. As shown in the first and second rows of the table, the CNN-based SED method with the mel-filterbanks from {Y} had a comparable RR to that with the CNN-based feature parameters from {Y}, while there was a tradeoff between MDR and FPR. This phenomenon was similar for the CNN-based SED methods with the CNN-based feature and mel-filterbanks applied to {$\hat{S}$}, as shown in the third and fourth rows of the table. On the other hand, the CRNN-based SED method with the mel-filterbanks provided better performance in RR, MDR, and AUC but slightly worse performance in FPR and AUC than that with the CNN-based feature parameters. However, as shown in the last row of Table 4, the proposed CRNN classifier with {$\tilde{S}$,$\tilde{D}$} significantly improved all the measures. This implied that the proposed CRNN classifier when combined with online noise learning was a better network architecture than the conventional CNN for both a signal processing-based and a neural network-based feature extraction approach.

### 4.6. Comparison of Computational Complexity

This subsection compares the computational complexity of both the conventional and the proposed SED methods. The measurements were (1) the number of parameters, (2) the average processing time to train each model per epoch for neural networks or the iteration of the expectation-maximization (EM) algorithm for the GMM–HMM, and (3) the average processing time for classifying a test signal one second long. To this end, all methods were implemented on a Linux-based workstation that consisted of an Intel Core i7, 64 GB of RAM with 11GB GTX-1080ti NVIDIA graphics. As shown in Table 7, the proposed CRNN had about twice the number of parameters of the conventional CRNN because its neural network was composed of two CNNs, as shown in Figure 6. Therefore, the average processing time to train the proposed CRNN was increased when compared to those for training the CNN and the conventional CRNN. Accordingly, the average processing time for testing the 1-s-long tunnel input noisy signal was about 11 ms, which was comparable to that of the conventional CRNN. This was because each CNN in the proposed CRNN was computed using a separate graphic processing unit (GPU), while the RNN of the proposed CRNN was less complex than that of the conventional CRNN. Consequently, since the processing time for the NTF-based source separation with online noise learning was measured at 588 ms, the proposed CRNN-based SED method had the processing time of 599 ms for the given test signal of one second. This implies that the proposed method could detect events under tunnel noise conditions in real time.

## 5. Conclusions

In this paper, a novel SED method was proposed for the robust detection of event signals in a tunnel environment. Unlike other tasks, SED in a tunnel environment had two difficulties: significant noise interference and very few sound event clips. To cope with these difficulties, the proposed method first used a preprocessing stage to adaptively separate a sound source signal from the input tunnel noisy signal with high variation, which was performed by applying online noise learning to the NTF-based source separation. In addition, a CRNN-based classifier was proposed to improve the detection accuracy by combining an event CNN and a noise CNN in the CRNN architecture.

In order to analyze the performance of the proposed SED method, two experiments were conducted using a publicly available audio events dataset for SED in a road environment and a tunnel environment dataset that was developed from real traffic sound recordings in a tunnel. In the first experiment with the road audio events dataset, the performance was compared with statistical SED methods such as SVM and GMM–HMM as well as neural network-based SED methods, such as CNN, the conventional CRNN, and the proposed CRNN. In addition, a SVM was constructed using one of the three different feature sets including MFCC features BoW, temporal and spectral features, and selected time and frequency features. It was shown that the SVM using selected time and frequency features provided the best performance of all SVMs. Next, the GMM–HMM, CNN, and CRNN were constructed using the mel-filterbanks from the noisy signal or the mel-filterbanks from the separated clean event sound by the NTF source separation with online learning. These classifiers were compared with the proposed CRNN with the NTF source separation with online learning. Consequently, it was shown that the proposed method gave the highest RR of 98.25%, the lowest MDR of 1.00%, and the highest AUC of 98.39%, while the FPR was comparable to that of the CNN. This was achieved due to the two CNNs in the proposed CRNN for modeling the event sound and noise separately. In addition, the performance contribution of the mel-filterbanks extracted from the proposed NTF-based online noise learning was compared with those of CNN-based features extracted from both noisy input and NTF without online noise learning. It was shown that the CNN-based based SED method with the mel-filterbanks provided comparable performance to that with the CNN-based feature parameters, while the CRNN-based SED method with the mel-filterbanks gave slightly better performance than that with the CNN-based feature parameters. This implied that the proposed CRNN-based SED method when combined with online noise learning was the best among the compared SED methods with both the mel-filterbanks and the CNN-based feature parameters.

Next, in the second experiment that used the tunnel environment dataset, the proposed CRNN was also compared with the GMM–HMM, CNN, and CRNN. Similar to the first experiment, the feature parameter set was extracted from either the noisy input signal or the event sound separated by the NMF source separation with online learning. From the performance comparison before and after applying the NTF source separation, it was shown that the CNN and CRNN after NTF provided a better RR, MDR, and AUC but a worse FPR than those before NTF. This was because the CNN and CRNN were trained using only the separated event audio without any consideration of the background noise. On the other hand, since the proposed CRNN-based SED method was constructed using both the event sound and noise separated from the NTF with online noise learning, the proposed CRNN outperformed other comparatives in all measurements by large margins. In particular, it reduced the recognition error rates by 47.40% and 28.56% when compared to the GMM–HMM-based and the conventional CRNN-based SED methods, respectively. Moreover, the FPR of the proposed CRNN was the lowest among all classifiers, which implied that the two CNNs for the event audio and noise mostly contributed to the performance improvement when compared to the conventional CRNN.

It should be noted that the proposed method can be applied to various SED applications, such as audio surveillance equipped with a CCTV in road noise environments for security and safety, scream detection integrated with a drone under severe mechanical noise conditions, or sound-based home surveillance. The proposed method can also be utilized for speech-based applications, such as speech and non-speech classification, speech-based emotion classification, and vocoder coding type classification through encoded speech.

In future work, to improve the performance of the proposed CRNN-based SED method, the incorporation of the NMF source separation into a neural network framework will be studied as in [31], where the challenge is determining how to characterize the online noise learning of NMF in a deep neural network. In addition, even though CNN-based feature extraction has been performed in this paper, further sophisticated investigations of the effect of such neural network-based feature extraction with online noise learning will be studied in detail.

## Figures and Tables

**Table 1:** Distribution of the MIVIA road audio events dataset [4].

Class	# Events	Duration
Tire skid (TS)	200	326.38 s
Car crash (CC)	200	522.5 s
Background noise (BN)	-	2732.0 s

**Table 2:** Distribution of the audio dataset for the development of SED in a tunnel environment.

Class	Training Set (Recorded)		Training Set (Generated)		Evaluation Set (Recorded)	
	# Events	Duration	# Events	Duration	# Events	Duration
Tire skid (TS)	54	120.55 s	311	383.45 s	39	109.88 s
Car crash (CC)	30	68.27 s	93	84.07 s	9	19.31 s
Background noise (BN)	-	5423.66 s	-	-	-	~ 48 h

**Table 3:** Configuration of network architectures of three different deep neural networks used for performance comparison.

Layer	Deep Neural Network		
	CNN	CRNN [32]	Proposed CRNN
No. of convolution layers	3	3	3, 3
No. of kernels	(8, 16, 32)	(8, 16, 32)	(8, 16, 32), (8, 16, 32)
Kernel size	(3, 3)	(3, 3)	(3, 3)
Pool size	(2, 2, 4)	(2, 2, 4)	(2, 2, 4), (2, 2, 4)
RNN layer	-	16 Bi-directional GRUs	16 Bi-directional GRUs
FC layer	Exists	Exists	Exists

**Table 4:** Performance comparison of the proposed and other SED methods evaluated on the MIVIA road audio events dataset.

Methods		Measures			
Features	Classifier	RR (%)	MDR (%)	FPR (%)	AUC (%)
MFCC features BoW * [4]	SVM	78.20	21	10.96	86.00
Temporal and spectral features * [4]	SVM	82.65	19	5.48	90.00
Selected time and frequency features * [23]	SVM	95.00	2.75	5.00	98.32
Mel-filterbanks from noisy signal {Y}	GMM–HMM [22]	67.75	32.00	29.76	82.90
	CNN	96.25	2.00	4.38	97.59
	CRNN [32]	96.00	3.25	3.06	97.01
Mel-filterbanks from NTF w/o online noise learning {$\hat{S}$}	GMM–HMM [22]	79.50	20.50	17.94	94.20
	CNN	94.00	2.75	3.94	96.56
	CRNN [32]	96.50	2.00	7.22	96.36
Mel-filterbanks from NTF with online noise learning {$\tilde{S}$}	GMM–HMM [22]	84.75	15.00	13.35	95.20
	CNN	96.00	2.50	2.40	97.45
	CRNN [32]	96.00	2.75	3.28	97.33
Mel-filterbanks from NTF with online noise learning {$\tilde{S}$,$\tilde{D}$}	Proposed CRNN	98.25	1.00	3.06	98.39

* Since the experimental setup using the MIVIA road audio events dataset was identical to the previous work in [23], the results of the star-marked methods indicated in the table were excerpted from [23].

**Table 5:** Performance comparison of the proposed and other SED methods evaluated on the real tunnel event dataset.

Methods		Measures			
Features	Classifier	RR (%)	MDR (%)	FPR (%)	AUC (%)
Mel-filterbanks from noisy signal {Y}	GMM–HMM [22]	69.81	30.19	88.68	69.11
	CNN	71.70	28.30	7.55	80.75
	CRNN [32]	81.13	18.87	11.32	82.66
Mel-filterbanks from NTF w/o online noise learning {$\hat{S}$}	GMM–HMM [22]	69.81	30.19	7.55	77.22
	CNN	79.25	20.75	41.51	64.68
	CRNN [32]	83.02	16.98	18.67	84.56
Mel-filterbanks from NTF with online noise learning {$\tilde{S}$}	GMM–HMM [22]	83.02	16.98	15.09	87.83
	CNN	83.92	16.07	17.57	85.87
	CRNN [32]	87.50	12.50	10.71	89.92
Mel-filterbanks from NTF with online noise learning {$\tilde{S}$,$\tilde{D}$}	Proposed CRNN	91.07	8.93	7.14	92.08

**Table 6:** Performance comparison of the CNN-based and CRNN-based SED methods with the CNN-based feature parameters and mel-filterbanks evaluated on the MIVIA road audio events dataset.

Methods		Measures			
Features	Classifier	RR (%)	MDR (%)	FPR (%)	AUC (%)
CNN-based feature from noisy signal {Y}	CNN	96.50	3.5	4.38	96.90
	CRNN [32]	95.25	4.75	5.03	96.09
Mel-filterbanks from noisy signal {Y}	CNN	96.25	2.00	4.38	97.59
	CRNN [32]	96.00	3.25	3.06	97.01
CNN-based feature from NTF w/o online noise learning {$\hat{S}$}	CNN	96.50	3.25	5.69	96.97
	CRNN [32]	96.00	3.50	5.03	96.09
Mel-filterbanks from NTF w/o online noise learning {$\hat{S}$}	CNN	94.00	2.75	3.94	96.56
	CRNN [32]	96.50	2.00	7.22	96.36

**Table 7:** Comparison of the number of parameters and processing time for training and testing the SED methods.

Item	SED methods			
	GMM–HMM [22]	CNN	CRNN [32]	Proposed CRNN
No. of parameters	9.6K	21K	34K	64K
Processing time for model training per epoch	4 s	5 s	8 s	12 s
Processing time per second of test signal ^+^	117 ms	2 ms	10 ms	11 ms

^+^ The NTF source separation with online noise learning required 588 ms, which was not counted for the processing time denoted in this table.
